# Amplitude of the SCN Clock Enhanced by the Behavioral Activity Rhythm

**DOI:** 10.1371/journal.pone.0039693

**Published:** 2012-06-28

**Authors:** Floor van Oosterhout, Eliane A. Lucassen, Thijs Houben, Henk Tjebbe vanderLeest, Michael C. Antle, Johanna H. Meijer

**Affiliations:** 1 Laboratory for Neurophysiology, Department of Molecular Cell Biology, Leiden University Medical Center, Leiden, The Netherlands; 2 Department of Psychology, Department of Physiology and Pharmacology, Hotchkiss Brain Institute, University of Calgary, Calgary, Canada; Vanderbilt University, United States of America

## Abstract

Circadian rhythms are regulated by the suprachiasmatic nucleus (SCN), a small structure at the base of the hypothalamus. While light effects on the SCN are well established, little is known of behavioral effects. This study elucidates direct modulating action of behavioral activity on the SCN by use of *in vivo* electrophysiology recordings, assessments of general locomotor behavior, and video-tracking of mice. The results show suppression of SCN neuronal activity by spontaneous behavior, the magnitude being dependent on the intensity, duration and type of behavioral activity. The suppression was moderate (32% of circadian amplitude) for low-intensity behavior and considerable (59%) for locomotor activity. Mild manipulation of the animals had reversed effects on the SCN indicating that different mechanisms are involved in the regulatory effect of spontaneous versus induced activity. The results indicate that exercise at the proper time of the cycle can boost the amplitude of the rhythm of the SCN clock itself. This has potentially beneficial effects for other rhythmic functions that are under the control of the SCN.

## Introduction

Mammals are equipped with a timekeeping system that enables anticipation and adaptation to environmental 24 h rhythms on earth. The master endogenous pacemaker is located in the suprachiasmatic nuclei (SCN) of the anterior hypothalamus [1]. Individual neurons of the SCN display cyclic expression of clock genes and protein products. The protein products lead to a rhythm in membrane excitability, a rhythm in electrical activity, and rhythmic release of humoral signals, giving rise to circadian rhythms in physiology and overt behavior [2]. Synchronization of the circadian system to the environmental day and night primarily relies on external light information, transmitted to the SCN via the retinohypothalamic tract [3].

While light is the most important cue for synchronization, the phase of the clock is also affected by the animal’s behavioral activity. Arousal or increased voluntary behavioral activity, brought on by a variety of stimuli, exert changes in the circadian clock that are evidenced by period changes or phase shifts in the overt functions [4]–[9]. For instance, wheel running activity is known to affect the period of the clock in a dose-dependent manner [10]–[14] and novelty-induced wheel running elicits phase shifts of the circadian activity rhythm [6], [15]. Also in humans, behavioral activity during the day accelerates phase resetting to new time zones [16]–[19] and physical exercise may have beneficial effects for circadian rhythm disorders related to dementia and aging [20]. A reduced rhythm amplitude is associated with aging and with a number of diseases, such as metabolic syndrome, depression, sleeping disorders and cardiovascular problems [21], [22]. It is of great importance therefore to understand the potential therapeutic benefit of physical activity and study the influence of behavioral activity on the circadian system.

*In vivo* recordings of electrical impulse frequency in the SCN of freely moving rats and hamsters have shown that behavioral activity is accompanied by an acute suppression of neuronal activity. While potentially intriguing, these studies were limited in their behavioral analysis. It remained unknown, for instance, which type of activity can suppress the SCN, as Yamazaki and coworkers studied exclusively running wheel activity [23], while Schaap and Meijer could not identify the type of behavior associated with SCN suppression [24]. By use of in *vivo* SCN electrical activity recordings, in combination with video-scored behavioral activity, we investigated the acute effects of various identified spontaneous behaviors on SCN electrical activity in the mouse. Additionally, we examined how the SCN responded to ‘induced’ rather than spontaneous behavior. We finally examined how behavioral activity at various times of the cycle affects the rhythm amplitude of the SCN clock.

## Results

### Suppression of the SCN Firing Rate

**Figure 1:**
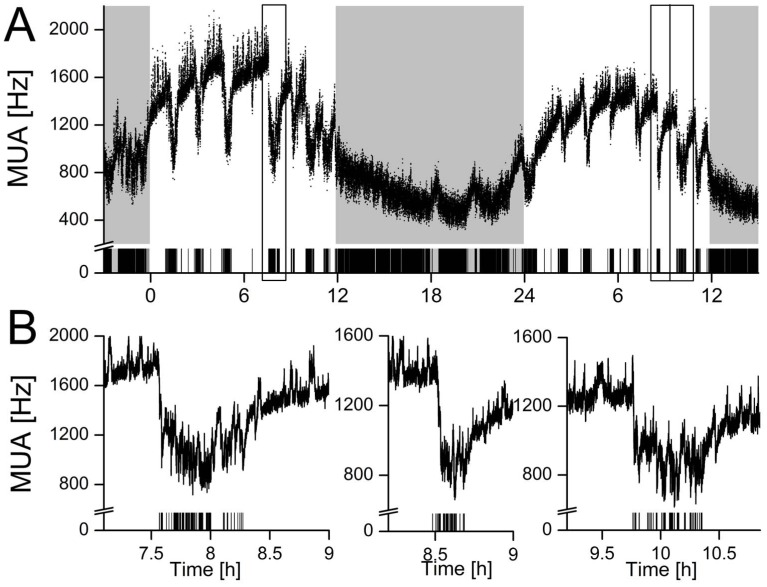
SCN electrical activity is suppressed during brief episodes of spontaneous behavioral activity. (**a**) Representative example of 48 h recording of SCN multiunit activity (MUA) in LD12∶12. Bin size is 2 s. The grey background indicates lights off. Lower bars represent simultaneous recordings of movement (all-or-nothing modus), as obtained by a passive infrared detector. (**b**) Expanded plots of SCN electrical activity during episodes of behavioral activity at mid-day as indicated by squared blocks in A. The X-axis shows *Zeitgeber Time* (hours) and the Y-axis shows SCN firing rate (Hz).

**Figure 2:**
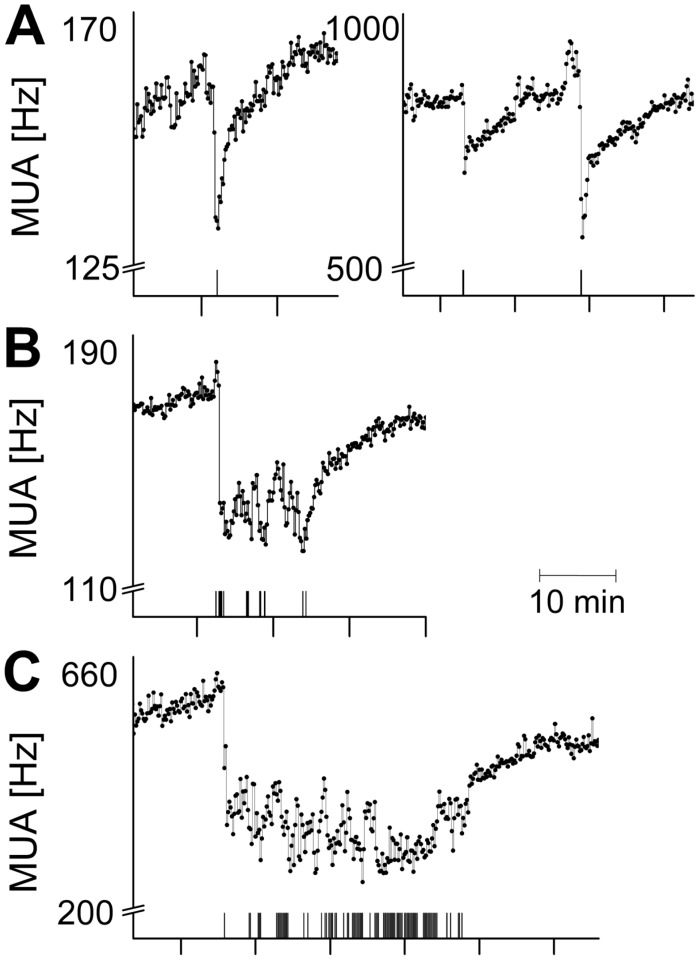
Time course of suppressions of SCN electrical activity during spontaneous behavioral activity. The start of behavioral activity is characterized by an acute drop of SCN firing rate. Decreased levels of SCN electrical activity are typically sustained throughout the duration of behavioral activity. Representative examples of different durations are shown. Behavioral activity and associated suppressed levels of SCN electrical activity last for approximately (**a**) 10 s, (**b**) 10 min, and (**c**) 30 min. Behavioral activity as detected by the passive infrared detector is plotted in the lower bar (all-or-nothing modus). Note that the PIR detector does not detect all behavioral activity which became apparent from the video recordings. Suppressions that lasted <1 min were observed in ∼20% of the cases, the occurrence of suppressions of 1–25 min was ∼ 65%, and the occurrence of long suppressions of >25 min was ∼15%. In each figure, the X-axis represents time (scale unit is given by the bar in the figure). Suppressions of all shown examples occurred between *Zeitgeber Time* (ZT) 3 and ZT10) under LD conditions. The Y-axis shows SCN firing rate (Hz). Bin size is 10 s.

**Figure 3:**
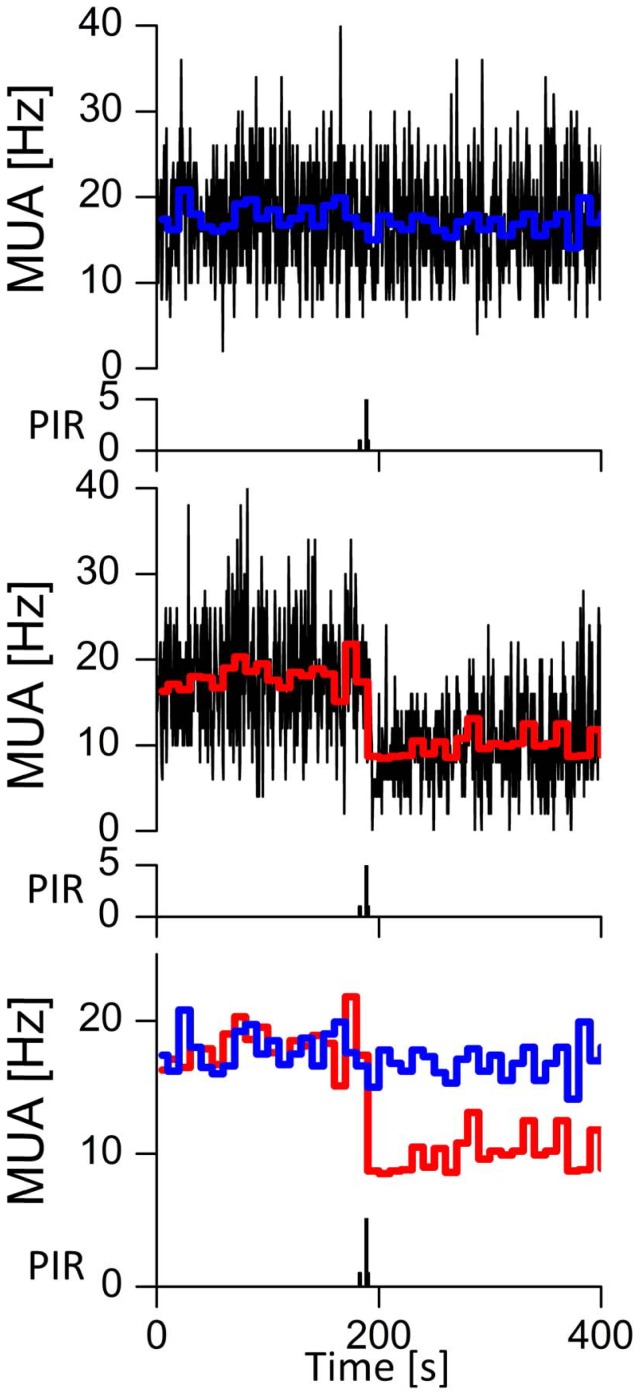
Electrical activity simultaneously recorded from two SCN sub-populations. Action potential thresholds were set off-line in such a way that an average firing frequency of 18 Hz was measured during baseline, in order to obtain approximately equal-sized populations of neurons. Bin size is 0.5 s, and lines in color represent the averaged firing frequency per 10 s. Spikes between excluding thresholds were counted, and revealed that the decreased firing rate during behavioral activity is apparent in some neurons (lower graph), while it is not present in others (upper graph). For comparison, the averaged firing frequencies are plotted in one graph below.

SCN electrical activity recordings were performed successfully in 14 animals and showed high levels during the subjective day (rest phase) and low levels during the subjective night (active phase). These rhythms were in anti-phase with the animal’s behavioral activity rhythm in all recordings. Behaviorally-induced suppression of the SCN firing rate was observed in 9 out of 14 animals (64%). Recordings in the other animals showed no response to behavioral activity. Instead, SCN neuronal activity of 4 out of these 5 mice showed responsiveness to light, while light-induced responses were not observed in the animals with behavioral suppressions. In the responsive mice, episodes of spontaneous behavioral activity were consistently associated with a decrease in SCN electrical activity (**Fig. 1**). Typically, at the start of behavioral activity, an abrupt drop in firing rate was observed. Firing frequencies remained suppressed for the duration of behavioral activity, and the electrical activity level gradually returned to baseline level after the animal had ceased its activity. Suppression of firing rates was observed in response to different durations of behavioral activity, even in response to episodes of activity as short as 10 seconds (**Fig. 2**). An analysis of the firing patterns based on spike amplitude histograms revealed that the observed suppression is present in specific subpopulations of the SCN, while in other subpopulations it is absent **(** **Fig. 3** **)**. No relation was observed with the site of the recording electrode. The suppression of SCN electrical activity occurred under LD cycles (observations from 9 animals), constant darkness (DD; observations from 7 animals) and in constant light (LL; observations from 2 animals). The magnitude of suppression reached values to up to 89% of the rhythm amplitude. The time course of recovery after an episode of behavioral activity followed an exponential pattern, and the time required to return to baseline levels was on average 15±4 min (range from 5 to 21 min), irrespective of the duration of behavioral activity and circadian time.

### Type and Intensity of Behavioral Activity

**Figure 4:**
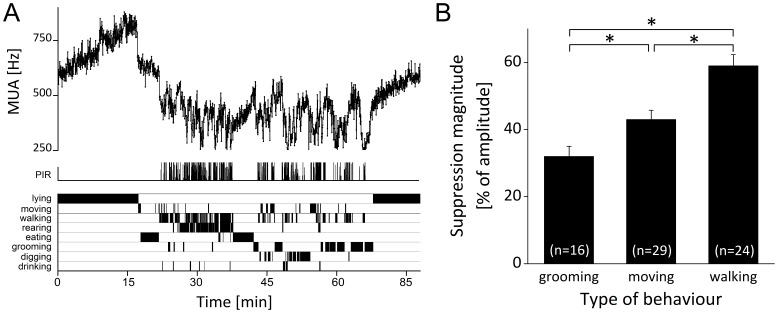
Relationship between type and intensity of behavioral activity and SCN electrical activity. (**a**) The SCN electrical activity profile as recorded throughout a full episode of behavioral activity (approximately 45 min). Lower bars represent the type of behavior as scored by video-observation. Behavioral activity as recorded by passive infrared detector is depicted at the bottom of the MUA trace in a graded scale. In this example, the initial behavior leading to a suppression of SCN activity is associated with moving, and is followed by eating which does not induce further suppression but keeps the SCN electrical activity at a reduced level. A further decrease of spike rate is induced when the animal starts locomotor behavior. While the suppression lasts for the full duration of the behavioral activity bout, gradual changes are associated with different types or intensities of behavior, and electrical activity gradually returns to baseline when the animal has ceased its behavioral activity. (**b**) The magnitude of suppression represented as a function of type of behavioral activity that initiated the suppression. Magnitude of suppression was significantly different between groups (*P<0.01, ANOVA with post hoc Bonferroni test). N-values are given between brackets. Error bars represent the standard error of the mean.

By use of video-analyses, different types of behavioral activity could be distinguished. Four animals had long video records that included examples of all behavioral subtypes. A number of clear suppressions (*n* = 69) were selected from these 4 animals for analysis (n = 11 were taken from LD conditions, n = 58 were taken from DD conditions). The SCN recordings and the accompanying video recordings were analyzed (**Fig. 4** **;** see **Video S1**). The magnitude of the suppression, expressed as a percentage of the amplitude of the circadian rhythm, ranged from 17% to 89%. In all cases, the animal had been lying or sitting quietly prior to the suppression. The type of behavior that was associated with the acute drop in electrical activity included one of the following behaviors: grooming, moving or walking. The magnitude of suppression of neuronal activity was related to the type of the initial behavior (**Fig. 4**). ‘Grooming’ behaviors inhibited the SCN firing rate by 32±3%, while ‘moving’ behaviors suppressed SCN firing by 43±3%. Walking produced the strongest suppression (59±3%) of electrical activity (*P*<0.01, ANOVA with post hoc Bonferroni test). While the behaviors ‘eating’ and ‘rearing’ were not found to initiate SCN neuronal suppression, these behaviors were often capable of maintaining electrical activity at reduced levels. Suppressed MUA levels as observed during non-intense behaviors, such as grooming, eating, drinking, and rearing, could be further decreased if they were followed by more intense behavior such as locomotor activity (**Fig. 4A**).

### Stimulated Behavioral Activity

**Figure 5:**
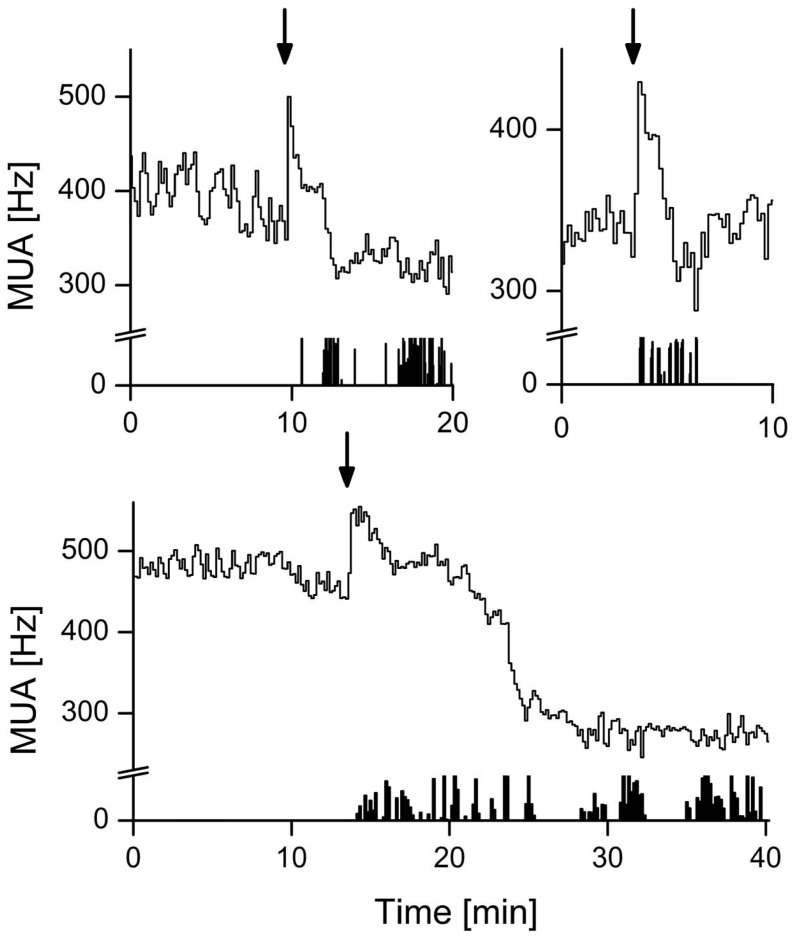
SCN electrical activity in response to mild disturbance of the animal’s rest during the day. The disturbance was established by making a small movement of the cage, without touching the animal. The timing of disturbance is given by the arrow. The increment of SCN discharge levels is acute, and returned to baseline quickly. In some cases the increment in electrical activity was followed by a suppression in electrical activity when behavioral activity continued (lower graph). It is possible that in these cases, induced activity had changed into motivated activity. Behavioral activity as recorded by passive infrared detector is depicted at the bottom of each MUA trace in a graded scale.

In contrast to spontaneous behaviors, mild disturbances of the animals produced an acute transient increase of the SCN firing rate that lasted for 10 s to 200 s (**Fig. 5**; see **Video S2**). The increment in SCN firing rate was observed in the same animals that showed inhibitory responses to spontaneous behavioral activity (Fig. 1). We had 3–5 observations (induced activity experiments) per mouse. Of the total number of observations, 40% were in LD and 60% in DD, and they were measured during the subjective day (CT/ZT 12–24).

During the increment in SCN firing rate, the animals often showed a freezing response, which was followed by locomotor activity. The elevated levels of SCN activity were either followed by a drop in electrical activity as compared to baseline, or they returned to baseline.

### Influence on Circadian Amplitude

**Figure 6:**
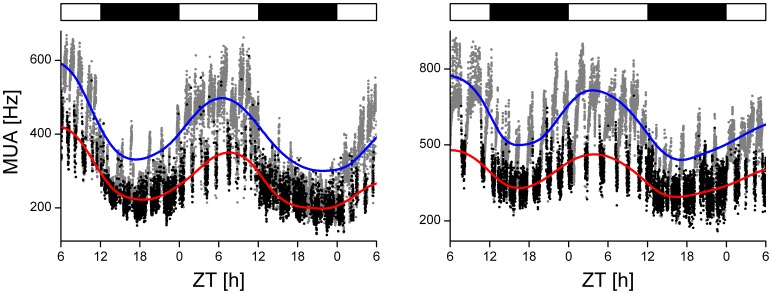
Circadian profile of SCN electrical activity in the presence and absence of PIR-recorded behavioral activity. To visualize the effect of behavioral activity on SCN rhythm amplitude, the passive infrared activity data are integrated in the electrical activity data: whenever passive infrared movement was detected during a 10 s recording bin, the number of SCN spikes counted in that bin is represented by a black dot. Grey dots show multiunit activity data points from bins when no movement was detected. Eye-fitted lines were drawn through grey dots (blue line) and black dots (red line) to illustrate the presence of a circadian rhythm in either profile. The LD12∶12 light cycle is indicated above the record (white, lights on; black, lights off).

Behavioral suppression occurred at all phases of the circadian cycle (based on 229 observations from 13 cycles from 2 animals in DD and in 280 observations from 16 cycles from 4 animals in LD; mean ± SEM = 19±1 observations per circadian hour). On average, the maximal occurring magnitude of neuronal suppression during the day was only slightly larger than during the night in all but one animal (averaged 47±1% (day; *n* = 266) vs. 43±1% (night; *n* = 233) respectively; *P*<0.05; ANOVA). We plotted the number of action potentials as a function of the time of the cycle, and distinguished between behaviorally active and inactive episodes of the mice. We found that clear circadian rhythms exist both in the absence and in the presence of behavioral activity (**Fig. 6**). The smoothed curves through the MUA rhythms revealed that the level of the rhythm was lowered in the presence of activity.

## Discussion

By use of stationary micro-electrodes, we measured the acute effects of behavior on the SCN and show that behavioral activity itself acts as a potent stimulus that modulates SCN pacemaker activity. The SCN electrical activity was strongly affected by the animal’s spontaneous behavioral activity level in an intensity- and duration-dependent manner. Electrical discharges were abruptly decreased upon the start of activity, and remained suppressed for the full duration of behavioral activity. By contrast, behavioral activity triggered by mild disturbance of the animal’s rest led to excitation instead of suppression of SCN activity. The spontaneous activity of the animals during the night resulted in a decrease of the trough level at night, while inactivity during the animals’ resting phase kept the peak of the SCN at high levels. Thus, a reciprocal interaction exists between overt behavioral activity and the rhythm amplitude of the central circadian pacemaker.

### Identification of Effective Behavioral Activity

Video recordings allowed for the identification of specific types of behavior that were able to induce suppression of SCN activity. We established the dependence of SCN neuronal suppression on the duration and intensity of activity. Surprisingly, ultra-short (<1 min) episodes of behavioral activity (**Fig. 2** and **4**) were capable of suppressing SCN activity to levels indistinguishable from those induced by longer activity periods. Furthermore, we found that non-intense types of behavior were capable of inducing substantial suppression (∼30%) of SCN electrical discharge levels. Notwithstanding, with more vigorous types of behavioral activity, the suppression of SCN activity became larger. The strongest modulation of SCN firing rates was associated with locomotor activity (∼60%; **Fig. 4**). When activity switched from one type to another, the electrical activity remained suppressed, but often the level of suppression changed, depending on whether the activity was more intense (further suppression), or less intense (**Fig. 4A**). The presence of behaviorally-induced suppression in mice is consistent with previous electrophysiological observations from rats [24], [25] and hamsters [23]. In hamsters, behavioral activity leads to suppressions in all recordings (Yamazaki et al, 2001) while in rats such suppressions were only observed in 5 out of 29 animals (Schaap and Meijer, 2001). These differences may relate to differences in the organization of input pathways, and to differences in phase shifting effects of behavior in these species. The present results indicate that more intense types of behavior lead to larger suppressions, with locomotor activity being the most potent modulator of electrical activity.

The neuronal suppression consistently occurred during spontaneous behavioral activity in the 9 responsive animals. However, it is not clear whether the two phenomena can be causally disentangled. We believe that the suppression of activity of the SCN is inextricably bound up with behavioral activity and may be caused by it. For one reason, in the isolated SCN *in vitro*, no spontaneous suppression of the SCN neuronal activity is observed while recording techniques are comparable (i.e. stationary MUA recordings) [26]. This indicates that input from, or interaction with, other brain areas is critical. Moreover, after termination of the behavioral activity, the electrical discharge recovered only slowly from suppression, while the behavioral activity was already fully arrested. Together, these findings suggest that physical activity leads to suppression of SCN electrical activity, most likely via activation of extra-SCN areas.

The direct suppressive effect of behavioral activity on SCN firing rate may relate to the finding that behavioral cues shift the circadian rest-activity cycle. The critical aspect of the behavioral stimulus causing these shifts is not fully identified. Previously, running wheel activity was considered essential to phase resetting of the clock. Indeed, a close correlation exists between hamster running wheel activity and the magnitude of a phase shift [15], and it had been shown that phase shifts were absent or attenuated when running wheel activity was blocked [8], [9]. However, sleep deprivation during the day with only minimal amounts of locomotor activity can also produce large phase shifts [4], and less clearly defined behavioral activity patterns, such as those induced by cage cleaning, lead to phase shifts, albeit of smaller magnitude. Our results can explain these behavioral findings; while locomotor activity has the largest effect on SCN activity, other types of behaviors can also acutely alter SCN firing rates, although to a lesser extent.

### Effects of Induced Versus Spontaneous Activity; Response Direction and Kinetics

When behavioral activity was evoked by mild disturbance of the animal, the observed responses of SCN neuronal activity were transient and excitatory. These responses were opposite to those induced by spontaneous behavior observed in the same animals (see **Fig. S1**). SCN neuronal discharge levels immediately increased in response to the stimulus and remained elevated for a maximum of 200 s **(** **Fig. 5** **)**. The kinetics of the recovery of the excitatory responses was different as compared to the recovery from suppressions. Following suppression, the recovery took remarkably long (approximately 15 min) and resembled an exponential function in accordance with previous results [24]. In contrast, the recovery from the excitation was fast. These findings raise questions on the potential underlying mechanisms.

The neuronal pathways involved in phase shifting by behavior are thought to originate from the raphe nuclei, which contains serotonin [27] (5-HT), and/or from the intergeniculate leaflet, which contains neuropeptide Y (NPY), GABA, enkephalin and neurotensin [3]. GABA_A_ activation in the SCN mimics the effects of non-photic manipulation on both circadian phase and clock gene expression [28]. Increased behavioral activity causes elevated levels of 5-HT [29] and NPY [30] in the SCN, and leads to increased c-Fos expression in hypocretin cells of the lateral hypothalamus [31]. Hypocretin application to coronal slices induces suppression of SCN neuronal activity with characteristic slow recovery [32]. Given the time course of the onset of the suppression, which is immediate upon behavioral activity onset, any of the inhibitory neurotransmitters could be involved, while the slow recovery at the end of the suppression would be consistent with the involvement of hypocretin or another neuromodulator.

While the procedure used to induce behavioral activity in our study could have activated a stress response, it is unlikely that a stress factor is causal to the change in SCN activity. First, the increase in SCN electrical discharge was instantaneous, whereas activation of a cortisol response would require more time. Furthermore, a similar approach for inducing behavior in hamsters has been shown to produce little cortisol response in the short term [33]. Finally, stress has little effect on the circadian system of rats and hamsters [33], [34], and corticosteroid receptors are absent in the SCN [35], [36]. Instead, the excitatory SCN response may involve the participation of arousal or anxiety systems [10], [37]. Neural structures that have been implicated in arousal responses include several nuclei in the diencephalon and brain stem, producing acetylcholine, histamine or norepinephrine and dopamine [38].

Elevations in firing rate may be due to excitatory stimulation of the SCN, as well as to a transient inactivation of hyperpolarizing signals (disinhibition). Thus, the involved neurotransmitter system could be excitatory or inhibitory. The analysis of subpopulations revealed heterogeneity within the recorded population. This suggests that incoming fibers reach part of the recorded area, and indicate the presence of refined but complex input organization. Similar heterogeneity in SCN responses has also been observed in response to photic signals [39].

When disturbed, mice in our experiment often initially froze but were alert. This behavior is usually associated with type 2 theta activity in the hippocampus, which is mediated by acetylcholine [40]. Acetylcholine is known to innervate the SCN from forebrain and brainstem cells [41]. It is possible that the cholinergic regions activating type 2 theta may also regulate SCN activity.

### Boosting Circadian Amplitude

**Figure 7:**
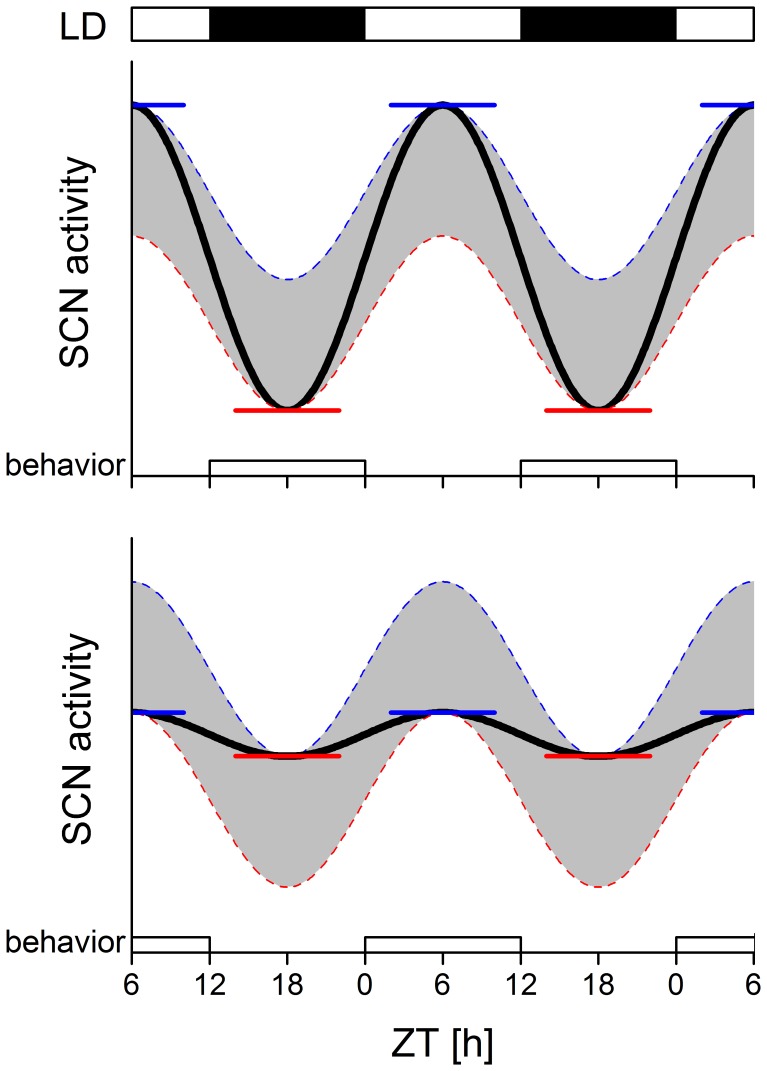
Consequence of night- versus day-time activity on SCN rhythm amplitude. Blue line represents the SCN activity in the absence of behavioral activity; red line represents the SCN activity in the presence of behavioral activity. Both lines are based on results shown in Figure 6. The black lines represent two extreme possibilities: (**a**) when behavioral activity is concentrated during the night and is absent during the day, the amplitude of the SCN electrical activity is maximal. (**b**) In contrast, when behavioral activity would occur during the day and rest occurs during the night, the SCN amplitude will be negatively affected and shows a reduced amplitude. Horizontal lines indicate peak and trough SCN activity levels. The LD12∶12 light cycle is indicated above the record (white, lights on; black, lights off).

The behaviorally-induced changes in SCN firing rate are superimposed on the SCN circadian rhythm and were found at all phases of the circadian cycle. As behavioral activity of nocturnal animals is concentrated during the night, suppression of SCN electrical activity is especially present during the night. As a result, the trough in SCN electrical activity is lowered. This finding indicates that the SCN rhythm amplitude can be boosted by behavioral activity during the animal’s active phase, i.e. the phase of the cycle where SCN electrical activity is low **(** **Fig. 7** **)**. Accordingly, the SCN pacemaker itself can be regarded as a node of a loop, in which SCN electrical activity and behavioral activity influence one another. When behavioral activity falls at the proper time of the cycle, i.e. when it occurs at night in nocturnal animals, the electrical activity of the SCN gains amplitude by a reduction of the trough level. When the animal is resting during the day, no suppressions will occur, keeping the peak of the SCN rhythm at a high level. Reversely, behavioral activity at uncommon phases of the circadian cycle (i.e. daytime for nocturnal animals) would result in lowered peak levels of SCN electrical activity, leading to reduced SCN amplitude (**Fig. 7**).

While the current results are obtained from nocturnal animals, it would be interesting to know how behavioral activity affects the clock in diurnal animals. Notably, the circadian profiles of 5-HT are linked to the animal’s 24 h activity-rest cycle rather than to the light-dark cycle, which implicates that these neuronal afferents cycle in anti-phase with the SCN in nocturnal animals, but is in phase in diurnal animals [42]. In humans, behavioral activity has a synchronizing effect on the circadian clock [8], [19], [43], [44], [45] and may have direct beneficial implications for health [20], [44]. For instance, in the elderly and in people suffering from Alzheimer’s disease or affective disorders, increased daytime exercise leads to improvement of sleep-wake rhythms, mood and performance [20], [45], [46], [47], [48], [49]. Transgenic mice with disturbed behavioral activity patterns show an improvement of behavioral activity with scheduled exercise [50]. Our results indicate that exercise may act by enhancement of the amplitude of the central pacemaker itself rather than through downstream mechanisms. The enhancement of rhythm amplitude of the clock could have beneficial consequences for other rhythms that are under control of the SCN. People living against their clock, such as shift workers, may end up with reduced rhythm amplitude of the central clock, by being active at the reversed part of their cycle. This would consequently have adverse effects on other rhythms that are under control of the SCN. Properly scheduled exercise may thus have beneficial effects for a broad range of physiological functions.

## Materials and Methods

### Ethics Statement

All experiments were performed under the approval of the Animal Experiments Ethical Committee of the Leiden University Medical Centre. Adult male C57BL/6 mice (Harlan) received electrode implantation under anesthesia with analgesics. The animal was connected to the recording system with a counterbalanced swivel system enabling the animal to move freely during the measurements.

### Micro-electrode Implantations

Procedures for *in vivo* recordings of multiunit activity from SCN neurons have been described previously for mice [51]. At a minimum age of 12 weeks (20–30 g), tripolar stainless steel micro-electrodes (Plastics One) were implanted in mice under Hypnorm/Dormicum anesthesia, using a stereotactic instrument. Two twisted electrodes (Polyimide-insulated; bare electrode diameter 0.125 mm) for differential recording were aimed at the SCN (coordinates: 0.46 mm posterior to bregma, 0.14 mm lateral to the midline, 5.2 mm ventral to the surface of the cortex, under a 5° angle in the coronal plane), and a third uncoated electrode was placed in the cortex for reference. The differential amplifier was based on the design of Yamazaki et al [23], and allows for recordings also during the animals’ behavioral activity.

### SCN Electrical Activity Recordings

After a recovery period of at least one week, the animal was individually housed in a temperature controlled recording chamber (22°C), where food and water were available *ad libitum* and where the mouse could move freely. The electrical signal was amplified, bandwidth filtered, and window discriminators were used to convert action potentials into digital pulses that were counted in 2 s bins (CircaV1.9 software, LUMC). Data were analyzed off-line by using OriginV7 software (OriginLab) and MATLAB (Mathworks Inc.).

### Behavioral Activity Recordings

Recording chambers (floor size 35×36 cm) were equipped with a drinking sensor and a Passive Infra-Red (PIR) movement detector positioned 30 cm above the floor of the cage. The experimental set-up did not allow for the recording of running wheel activity. Behavioral activity profiles were recorded simultaneously with electrical activity in similar time bins (2 s). The presence of activity as detected by the passive infrared detector is represented by a all-or-nothing modus (*e.g.* **Figs.** **1** **and** **2**), or by a graded scale (*e.g.* **Figs. 3** **,** **4** **, and** **5**). After surgery, 14 animals were kept on LD12∶12, and after several cycles the animals were released into constant darkness (DD) or constant light (LL). As a routine, the presence of a light-induced response in SCN electrical activity was tested by 5-min light exposure during the subjective night (Naturalite light bulb; 76 µW/cm^2^ = 105 lux). Activity measurements were verified and inspected at more detailed levels by video-records provided by an infrared-sensitive camera (AXIS 221 Network Camera, NL) that was placed in front of the cage. The cage was illuminated with infrared LEDs to provide a clear image for the infrared-sensitive camera in the dark. Type and intensity of spontaneous behavioral activity were analyzed off-line. In order to test the SCN responsiveness to ‘forced’ activity, behavioral activity was induced in 6 animals by mild disturbance of the animal’s rest. For this test, we used only animals that showed suppressions of SCN impulse frequency with spontaneous behavioral activity. Procedures included either the production of some noise in the proximity of the animal’s cage or small movements of the floor of the cage.

### Data Analysis – SCN Electrical Activity

Electrical activity data were smoothed in MATLAB (Mathworks Inc.). The smoothing parameter was set to the lowest value yielding a single peak and through per circadian cycle. Rhythm amplitudes were determined by measurement of the difference between peaks and troughs. The magnitude of the behaviorally-induced neuronal suppression was defined as the difference between baseline electrical activity and the average electrical discharge during the suppression. The baseline level was defined as the mean discharge rate during the last 2 minutes before the suppression. The magnitude of the suppression was expressed as a percentage of the amplitude of the SCN rhythm. Single factor ANOVA analysis was performed to determine whether the electrical activity level during the animal’s behavioral activity was significantly inhibited as compared to baseline level. The magnitude of the suppression was studied as a function of circadian time in 6 animals over a period of 3–7 cycles. Two-way ANOVA with post-hoc Bonferroni test was used to test for differences between day (CT0-12) and night (CT12–24) and *P*<0.05 was considered to be statistically significant.

### Data Analysis – SCN Subpopulation Activity

To enable a more detailed analysis of SCN sub-population activity, the electrical signal was digitized at 25 kHz using Spike2 hardware and software (Cambridge Electronic Design). Off-line, the data were imported into MATLAB as ‘waveform data’ using parts of the sigTOOL SON Library (http://sigtool.sourceforge.net). A baseline recording (100 s before behavioral activity occurred) was used to create a spike amplitude histogram. On the basis of this amplitude histogram, thresholds were set in such a way that the average number of counts within each threshold window was equal. Threshold windows were non-overlapping and started above noise level. Action potentials were counted within each step of set thresholds. The measurement of SCN sub-populations on the basis of baseline electrical activity levels leaves the neuronal response after baseline as a free parameter, and enables comparisons between different, approximately equal-sized, populations of neurons.

### Video-analysis

By video-analysis, the following behavioral activities were scored in 1 s bins: digging, drinking, eating, grooming, lying, moving, rearing, sitting, and walking. For the analysis, we used the data of 4 animals from which we had long recordings that included all types of scored behavior. With respect to the protocol for ‘induced’ behavioral activity, video was recorded to assure the induction of behavioral activity and to qualitatively analyze the type of behavioral activity following the mild disturbance procedure.

### Histology

To verify the electrode location at the end of the experiment, animals received an overdose of barbiturate, after which a small electrolytic current was passed through the recording electrode to deposit iron at the electrode tip. The brains were collected and immersed in a potassium ferrocyanide containing fixative solution which caused the iron deposits in the brain to turn blue. Brains were sectioned coronally (10 µm slices) and stained with neutral-red for microscopic reconstruction of the recording site (see **Fig. S2**). Recordings from outside the SCN were excluded from analysis.

## Supporting Information

Figure S1 **Illustration of SCN suppression and SCN excitation in the same animal.** Representative example of a trace showing suppression of SCN electrical activity in response to spontaneous behavioral activity (panel **A**). The occurrence of suppression was set as a criterion for testing the SCN response to stimulated behavior, which appeared to be excitatory as is shown by the recording trace in panel **B** (and see **Figure 5**). Recordings from **A** and **B** were depicted during the subjective day. Bin size is 2 s. Lower bar represents the animaĺs spontaneous movements as recorded by a passive infrared detector. Note that the PIR sensor does not detect all behavioral activity which became apparent from the video recordings. The timing of the disturbance of the animal’s rest is given by the arrow. X-axis represents time (min), Y-axis represents the SCN multiunit activity (Hz).
(TIF)

Figure S2 **Histological sections containing the SCN and electrode track.** Microscopic images of coronal brain sections from three mice showing the location of the electrode, which is marked by a blue spot and/or mechanical track damage. The suprachiasmatic nuclei are visible as clusters of densely stained cells, embedded in the optic chiasm at both sides of the third ventricle.
(TIF)

Video S1 **Suppression of SCN activity by spontaneous behavior.** The animal is lying quietly before the start of the video fragment. Video recording starts at time 20 s. The electrical impulse frequency of the SCN is depicted on the y-axis. The movements as detected by the passive infrared detector (PIR) are indicated below the electrical activity trace. At the bottom, the following behaviors were scored: lying, moving, grooming, sitting, walking and eating.
(MOV)

Video S2 **Excitation of SCN activity by induced behavior.** The animal was disturbed and activated by a movement of the cage at time 60 s. This resulted in a temporal increment of SCN electrical activity.
(MOV)
