# Supplementary figures and images for: Amplitude of the SCN Clock Enhanced by the Behavioral Activity Rhythm
Source: PLoS One. 2012 Jun 28;7(6):e39693. doi: 10.1371/journal.pone.0039693 (PMC3386260; doi:10.1371/journal.pone.0039693)

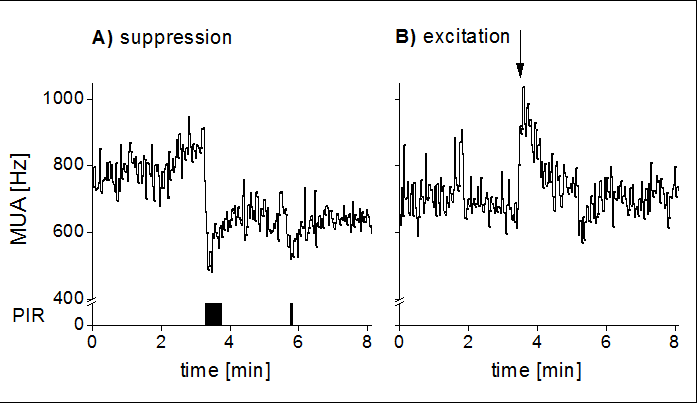

Supplement: Figure S1 — Illustration of SCN suppression and SCN excitation in the same animal. Representative example of a trace showing suppression of SCN electrical activity in response to spontaneous behavioral activity (panel A). The occurrence of suppression was set as a criterion for testing the SCN response to stimulated behavior, which appeared to be excitatory as is shown by the recording trace in panel B (and see Figure 5 ). Recordings from A and B were depicted during the subjective day. Bin size is 2 s. Lower bar represents the animaĺs spontaneous movements as recorded by a passive infrared detector. Note that the PIR sensor does not detect all behavioral activity which became apparent from the video recordings. The timing of the disturbance of the animal’s rest is given by the arrow. X-axis represents time (min), Y-axis represents the SCN multiunit activity (Hz). (TIF) [file pone.0039693.s001.tif]

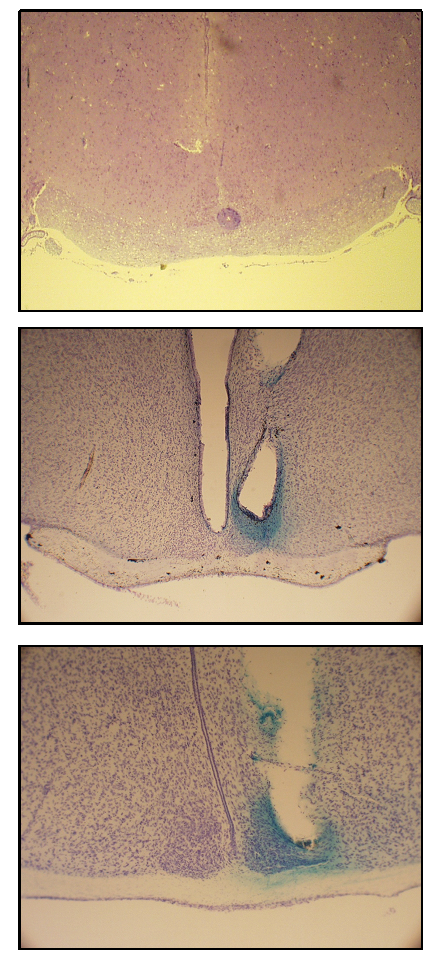

Supplement: Figure S2 — Histological sections containing the SCN and electrode track. Microscopic images of coronal brain sections from three mice showing the location of the electrode, which is marked by a blue spot and/or mechanical track damage. The suprachiasmatic nuclei are visible as clusters of densely stained cells, embedded in the optic chiasm at both sides of the third ventricle. (TIF) [file pone.0039693.s002.tif]
